# Evaluation of an English language phoneme-based imagined speech brain computer interface with low-cost electroencephalography

**DOI:** 10.3389/fninf.2023.1306277

**Published:** 2023-12-18

**Authors:** John LaRocco, Qudsia Tahmina, Sam Lecian, Jason Moore, Cole Helbig, Surya Gupta

**Affiliations:** ^1^Wexner Medical Center, The Ohio State University, Columbus, OH, United States; ^2^Department of Electrical Engineering, The Ohio State University, Columbus, OH, United States

**Keywords:** EEG, electroencephalography, covert speech, imagined speech, brain-computer interface, BCI

## Abstract

**Introduction:**

Paralyzed and physically impaired patients face communication difficulties, even when they are mentally coherent and aware. Electroencephalographic (EEG) brain–computer interfaces (BCIs) offer a potential communication method for these people without invasive surgery or physical device controls.

**Methods:**

Although virtual keyboard protocols are well documented in EEG BCI paradigms, these implementations are visually taxing and fatiguing. All English words combine 44 unique phonemes, each corresponding to a unique EEG pattern. In this study, a complete phoneme-based imagined speech EEG BCI was developed and tested on 16 subjects.

**Results:**

Using open-source hardware and software, machine learning models, such as k-nearest neighbor (KNN), reliably achieved a mean accuracy of 97 ± 0.001%, a mean F1 of 0.55 ± 0.01, and a mean AUC-ROC of 0.68 ± 0.002 in a modified one-versus-rest configuration, resulting in an information transfer rate of 304.15 bits per minute. In line with prior literature, the distinguishing feature between phonemes was the gamma power on channels F3 and F7.

**Discussion:**

However, adjustments to feature selection, trial window length, and classifier algorithms may improve performance. In summary, these are iterative changes to a viable method directly deployable in current, commercially available systems and software. The development of an intuitive phoneme-based EEG BCI with open-source hardware and software demonstrates the potential ease with which the technology could be deployed in real-world applications.

## 1 Introduction

Difficulties in communication greatly reduce the quality of life of paralyzed and physically impaired individuals. Electroencephalographic (EEG) brain–computer interfaces (BCIs) offer a potential communication method for these people because they do not require invasive surgery or physical device controls. Although virtual keyboard protocols are well documented in EEG BCI paradigms, the P300 speller and steady-state visually evoked potentials (SSVEPs) are visually taxing and fatiguing. Motor imagery can be hard-coded to specific keys or buttons; however, this requires extensive data training and time-consuming encoding of multiple specific gestures. In a machine learning classifier, the covert or imagined speech BCI paradigm encodes specific EEG patterns of imagined thought to discrete outputs. Linguistic core components, phonemes, have been reported as separable in an EEG pattern (Suyuncheva et al., 2020). All English words are combinations of 44 unique phonemes, each corresponding to a unique EEG pattern. Therefore, using a phoneme-based covert speech EEG BCI may be the least visually taxing and most intuitive method of converting thought to speech. Prior covert speech systems have used expensive research and commercial headsets at higher sampling rates (Panachakel and Ramakrishnan, 2021; Lopez-Bernal et al., 2022; Shah et al., 2022). If a more capable covert speech BCI could be successfully developed using a commercial EEG headset, its accessibility would be significantly increased. In this study, an EEG BCI using 44 English language phonemes was implemented with low-cost and open-source hardware, and the software was evaluated on 16 human subjects.

### 1.1 Background

Communication can be challenging for paralyzed and physically impaired individuals. Prior studies on invasive implants, specifically for communication, have resulted in health complications, such as tissue scarification and implant rejection. Imagined speech BCIs offer a potential alternative; however, previous studies have used expensive tools, such as multichannel EEG or MRI scanners (Tang et al., 2023). The use of specific letters, words, or syllables has succeeded in imagined speech BCIs; however, the use of phonemes has been limited (Shah et al., 2022).

### 1.2 EEG BCI

For decades, non-invasive EEG BCI systems have been used in research and recreation (Di Flumeri et al., 2019; Kübler, 2020). While surface EEG is noisier than invasive recordings, EEG BCI systems do not cause the potential complications of invasive, implanted devices (Guenther et al., 2009). Low-cost EEG systems have become increasingly common in applications, such as medical devices, commercial products, and hobbyist projects (Cardona-Alvarez et al., 2023). EEG BCI systems use reliably repeatable patterns of electrophysiological activity. The specific visual or auditory stimuli used to evoke the electrophysiological activity consists of the paradigm. Examples include the P300 speller using flashing rows and columns and motor imagery consisting of cued imagined physical gestures (Tang et al., 2022). The P300 speller and SSVEP have been used as virtual keyboards to enable the letter-by-letter composition of a message (Capati et al., 2016). However, owing to their flashing lights, such paradigms are visually taxing and challenging to use for extended periods (Allison et al., 2010). Motor imagination BCIs involve using the distinctive EEG of separate imagined physical gestures as device inputs (Lakshminarayanan et al., 2023). Motor imagery has been used for virtual keyboards and other applications, but the most active brain region is typically the brain’s motor cortex. In contrast, imagined speech involves the brain’s language forming areas and regions (Lopez-Bernal et al., 2022). While activity in brain regions can overlap, imagined speech is distinct from imagined gesture-based EEG BCI.

### 1.3 Imagined speech

Imagined speech, also called covert speech, is a BCI protocol that uses imagined verbal utterances (Kim et al., 2013; Sereshkeh et al., 2017a,b). Neural control of speech is a more complex, diffuse process than motor action. A prior researcher’s invasive implant was designed to directly interface with the verbal and linguistic networks of the brain (Panachakel and Ramakrishnan, 2021; Shah et al., 2022). An alternative approach involves functional magnetic resonance imaging (fMRI) to reconstruct entire words and sentences. Owing to its accessibility and low cost, most imagined speech implementations have used EEG. However, these implementations are impractical for daily use (Panachakel and Ramakrishnan, 2021; Shah et al., 2022).

Prior EEG imagined speech BCIs have involved several constraints. The first implementations of imagined speech were conducted on research or medical EEG systems with gel electrodes and higher numbers of channels requiring long setup times (Jahangiri and Sepulveda, 2019; Panachakel and Ramakrishnan, 2021; Lopez-Bernal et al., 2022). The use of visual and auditory stimuli to evoke EEG responses can distract from immediate tasks. BCI illiteracy occurs with imagined speech, as with other BCIs. The breadth of disjointed linguistic components has also constrained BCIs. Some studies have focused on complete words, syllables, sentences, or phonemes (Jahangiri and Sepulveda, 2019; Panachakel et al., 2021). Because every language comprises multiple phonemes, no imagined speech EEG study has examined all the constituent phonemes of a language. An EEG BCI not requiring visual and auditory stimuli precludes the need to look at a distracting screen. As phonemes are the core component of any language, their use could make imagined speech BCIs significantly more intuitive.

### 1.4 Phonemes

Phonemes are the basic components of every spoken language, and English has 44 separate phonemes (Ariki, 1991). Previous research has identified the EEG-based separability of phonemes (Panachakel et al., 2021). The use of EEG corresponding to unique phonemes has been successfully reported in an imagined speech BCI (Suyuncheva et al., 2020; Panachakel et al., 2021). However, no existing EEG BCIs have utilized all English-language phonemes. The successful demonstration of an offline BCI EEG corresponding to all phonemes could demonstrate the viability of this concept. Training and testing such a system on data collected on a dry-electrode hobbyist headset would significantly improve the accessibility of non-invasive, thought-to-speech systems.

## 2 Methods and materials

### 2.1 Overview

Deploying a phoneme-based imagined speech EEG BCI requires the correct stimuli, data acquisition, feature extraction, and classification. Each potential user was asked to observe and listen to a stimuli presentation while the EEG was recorded (Panachakel and Ramakrishnan, 2021). An EEG headset and acquisition software were used. Feature extraction required knowledge of the most reliable EEG features for the imagined speech of each phoneme. A classifier model that would provide accurate results and that did not overfit was required for data classification. Overall, the BCI system necessitated using the most reliable aspects of prior studies (Lopez-Bernal et al., 2022).

### 2.2 Participants

**Figure 1:**
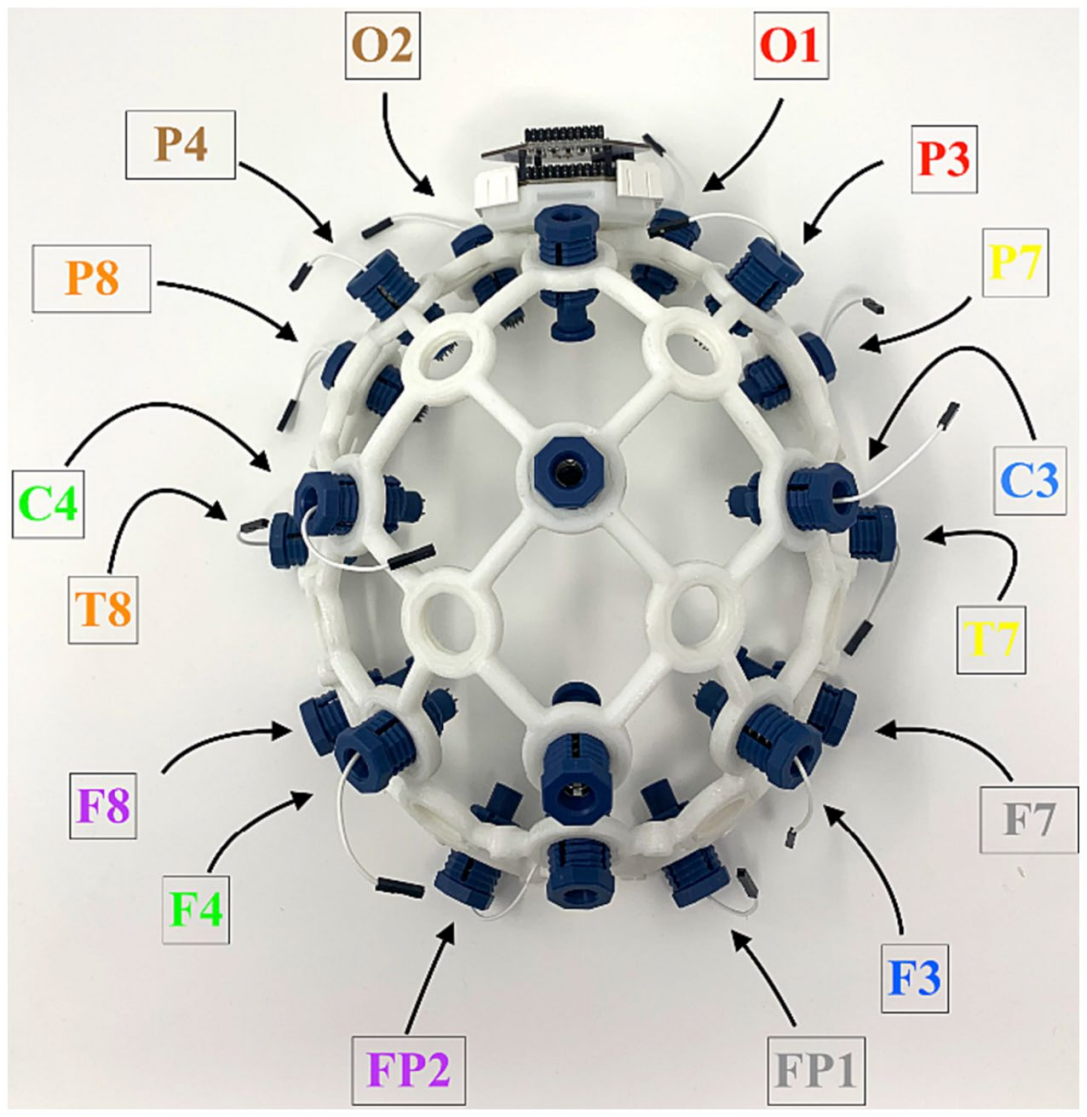
Electrocephalographic headset used for data acquisition, shown with 16 electrodes in 10–20 International System and an OpenBCI Cyton board.

A total of 16 participants were recruited through word of mouth and flyers. All participants consented to the experiment, which was approved by IRB 2023H0194. The average age was 27.3 ± 1.2 years, and the participants comprised 4 females and 12 males. Participants had normal hearing and normal or corrected vision. Most participants were native English speakers, with 5 non-native English speakers who demonstrated functional proficiency on the standardized exams required for university admission. After providing consent, participants were positioned at least 24 inches from a display monitor. The participants put on the EEG headset shown in Figure 1 and attached the reference electrodes. To begin the first session, instructions were displayed on the screen, and recording began.

### 2.3 Stimulus presentation

**Figure 2:**
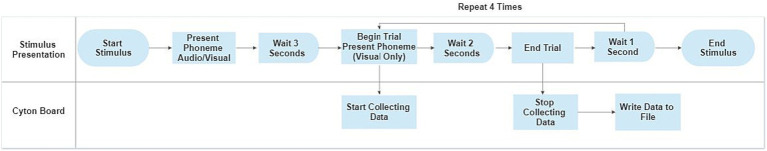
System operation diagram for each session.

All software tasks were performed using Python (Amemaet, 2021). Prior studies in imagined speech have used auditory and visual stimuli to generate training data (Panachakel and Ramakrishnan, 2021; Shah et al., 2022). However, the repeated use of auditory stimuli would result in distractions. Therefore, a combination of visual and auditory stimuli was used for each phoneme. The presentation order of each phoneme was randomized at the start of each training session. Participants were instructed to imagine saying each phoneme. Each phoneme presentation had an identical format. The chronological sequence of stimuli is shown in Figure 2.

Data recording for each phoneme included a demonstration and five separate trials. The demonstration presented a white screen with black characters. The sequence is shown in Figure 2. The phoneme was shown for 2 s. An auditory pronunciation of the phoneme was played once, at the start of each phoneme. A screen with the word ‘wait’ was presented as an interval for 1 s. The phoneme was displayed again for 2 s, and the participant was instructed to think of speaking during this time. The character representation for each phoneme was then put on screen for 2 s, followed by a ‘wait’ screen for 1 s. Participants were instructed to stop imagining during the ‘wait’ interlude screen, and the corresponding EEG data from these interlude segments was not used. This sequence was repeated five times without auditory feedback for five trials per phoneme. Each session included five trials of 44 phonemes in random order. Each session lasted approximately 40 min, and three sessions were recorded. If all sessions could not be recorded, as much data as possible was acquired. Data were discarded if a complete set of phonemes was not successfully recorded.

### 2.4 Data acquisition

To facilitate integration with open–source hardware and software, data were acquired using an OpenBCI Cyton board and an Ultracortex Mark IV headset (OpenBCI Foundation, New York). Data from 16 EEG channels were acquired at 250 Hz. Data collection was assisted and timestamped using a Python script. As shown in Figure 1, the 10–20 International System electrode channels used included Fp1, Fp2, F7, F3, F4, F8, T3, C3, C4, T4, T5, T6, P3, P4, O1, and O2 (Trans Cranial Technologies, 2012).

Each trial was saved as a separate file. The phoneme, trial, and participant numbers were included in the name. If a trial was not successfully timestamped, it was excluded from future processing. At least two successfully timestamped trials per phoneme per participant were required to include the participant in the dataset. Feature extraction and classification were performed offline following data acquisition.

### 2.5 Feature extraction

The selection of feature types was based on prior studies, primarily the spatiotemporal features and amplitude (Torres-García et al., 2016). Each file was loaded, and all trials within each file were separated by timestamp. Each file included approximately 2 s of EEG data. EEG data from each channel was separated into two windows of 1 s each, with each processed separately. A non-overlapping, 1-s window length used due to prior work (Panachakel and Ramakrishnan, 2021). A feature extraction process was conducted on time series data from each window. If the total amplitude of the recorded values was more than three standard deviations away from the baseline, the averaged EEG amplitude, of that session, it was rejected as an artifact. Otherwise, the signal was bandpass filtered between 0.1 and 125 Hz with a 4^th^-order Butterworth filter. Resonant frequencies of the overhead power, 60 Hz, were also removed. Then, the temporal average was calculated, a feature successfully used in prior imagined speech BCIs. Afterward, the percent intensity of each window for 99.95% was calculated. Reflecting other EEG studies, several average power spectral densities bands for the major EEG bands [delta (1–4 Hz), theta (5–8 Hz), alpha (8–12 Hz), beta (13–30 Hz), and gamma (30–100 Hz)] of each feature were calculated using Welch’s method (LaRocco et al., 2014, 2020). The mean powers of the lower and higher frequency ranges of each EEG band were also calculated (e.g., 8–10 Hz for the lower range of the alpha band). The extracted features included non-normalized spectral features, and those scaled relative to the total spectral power.

**Figure 3:**
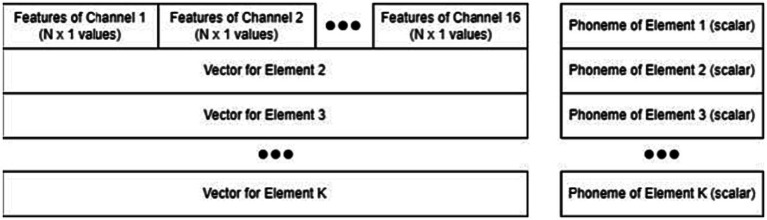
Structure of the feature matrix and label vector, including stacked feature vectors and individual phoneme labels.

As shown in Figure 3, the 35 features from each channel were concatenated into a longer 1D feature vector of 560 elements for each sampling window. Then, the feature vectors of the first and second windows were concatenated into a single array: a 1D row of 1,120 elements. This feature vector was calculated for each successfully timestamped trial. There were three sessions, 44 phonemes, and five trials per phoneme, and the total number of trials per participant was 660. Therefore, the final feature matrix was 660 trials (rows) by 1,120 features (columns).

The structure of the data and label vectors is shown in Figure 3. If the number of successful trials or sessions was smaller, the feature matrix was similarly reduced in length. Each participant had a feature matrix.

### 2.6 Data classification

Two primary data classification methods were used: intrasubject classification and intersubject classification. Intrasubject classification determined a participant’s potential viability for an imagined speech BCI. A low classification score, corresponding to a low accuracy, F1 score, or area under the receiver operating characteristic curve (AUC-ROC), implied poor data quality. Intersubject classification determined the generalizability of the imagined speech EEG BCI system. If a classifier model could reliably classify EEG features from several subjects, an imagined speech EEG BCI may simply require a sufficient amount of data. The average distance between events and non-events (ADEN)-based feature selection, based on two statistical weighting methods, was used to determine the most important features in each configuration (LaRocco et al., 2014).

ADEN was a supervised feature selection method, used to select the top three to six unique features for each run, based solely on the available training data. The features for each class were averaged. A combination of a *z*-score transform and Cohen’s *d* were used to scale both, followed by taking the absolute value of the difference. The largest value corresponded to the greatest average distance between two classes, the second largest value to the second greatest average distance, and so on. The top three to six features, based on magnitude of the scaled distance, were retained for further use on the validation data (LaRocco et al., 2014).

Overfitting was a potential concern as there were 16 channels and potential noise. Measures independent of class distribution and indicative of low false positive rates, such as F1 and AUC-ROC, were prioritized over classifier accuracy to account for potential overfitting. Consequently, traditional machine learning models were used instead of deep learning. Based on prior algorithms used in similar BCIs, three separate algorithms were used: linear discriminant analysis (LDA), linear-kernel support vector machines (SVM), and k-nearest neighbors (KNN) (Shah et al., 2022). In each case, data were randomly divided into four blocks. Each classification problem was structured as a one-versus-rest for each of the 44 phonemes, and the categories were balanced based on small sample size technologies (Sereshkeh et al., 2017a; Jahangiri and Sepulveda, 2019). Training and testing datasets were kept equivalent to ensure balanced class distributions. Each phoneme-specific classifier used 4-fold leave-one-out cross-validation (LOOCV) to ensure the results were reliable. The fourth block was withheld for validation in each system configuration. The one-versus-rest configuration has reached a reported accuracy of 96.4% in prior studies, and a similar system was adapted to real-time use as a synchronous BCI (Sereshkeh et al., 2017a; Jahangiri and Sepulveda, 2019). The averaged accuracy, F1, and AUC-ROC scores for each system and phoneme were averaged together. All models were run for both intrasubject and intersubject classification.

### 2.7 Performance assessment

Because an instinctive imagined speech BCI could significantly improve the performance of electronic commands and messages, the information transfer rate (*ITR*) for each system configuration was calculated using Eq. 1 (Blankertz et al., 2006).


(1) $$ITR\left(\frac{\text{bits}}{\text{trial}}\right)=\log_{2}\left(N\right)+P\times \log_{2}\left(P\right)+\left(1-P\right)\times \log_{2}\left(\frac{1-P}{N-1}\right)$$

In Eq. 1, ITR is expressed as bits per trial. The performance of a BCI is directly related to the number of classes (N) and accuracy (P). In the implemented BCI, the value for N is equal to the number of phonemes, 44. Although a low ITR can have direct applications, the maximum ITR of each system configuration was calculated to model the highest possible performance (LaRocco and Paeng, 2020). To simplify the calculation, a sampling window of 1 s was assumed, following the data acquisition protocol. Equation 2 was used to convert ITR to bits per minute.


(2) $$ITR\;\left(\frac{\text{bits}}{\min}\right)=ITR\left(\frac{\text{bits}}{\min}\right)*1\left(\frac{\text{trial}}{\text{seconds}}\right)*60\;\left(\frac{se\text{conds}}{\min}\right)$$

Classifier performance is the key parameter for a high ITR calculation. Based on prior performance, it was hypothesized that LDA would perform the highest on average in terms of accuracy and F1 score (Jahangiri and Sepulveda, 2019). Based on previous studies, it was hypothesized that the top features in each case would be the spectral band power and average mean amplitude, as calculated from electrodes on the top and front (Torres-García et al., 2016; Panachakel and Ramakrishnan, 2021). Electrodes, such as C4, F3, and F7, in the 10–20 International System have been previously related to EEG signatures of phonemes owing to their proximity to speech-forming areas of the brain, such as Broca’s region (Trans Cranial Technologies, 2012; Pan et al., 2021; Wang et al., 2021; Lopez-Bernal et al., 2022).

## 3 Results

### 3.1 Overview

Classifier performance was examined. The first analysis examined intrasubject classification. The second analysis examined intersubject classification to determine if training data could be generalized across subjects. The third analysis assessed the determination of the features and electrodes corresponding to the most reliable separation between phonemes. The ITR of the most relevant results was calculated for each phase.

### 3.2 Intrasubject classification

For intrasubject classification, the highest-performing classifier for F1 and AUC-ROC was KNN. KNN reached a mean accuracy of 97 ± 0.024%, a mean F1 score of 0.55 ± 0.03, and a mean AUC-ROC of 0.69 ± 0.003. LDA achieved a mean accuracy of 98 ± 0.01%, a mean F1 score of 0.50 ± 0.024, and a mean AUC-ROC of 0.62 ± 0.001. SVM achieved a mean accuracy of 98 ± 0.003%, a mean F1 score of 0.50 ± 0.03, and a mean AUC-ROC of 0.50 ± 0.002.

**Figure 4:**
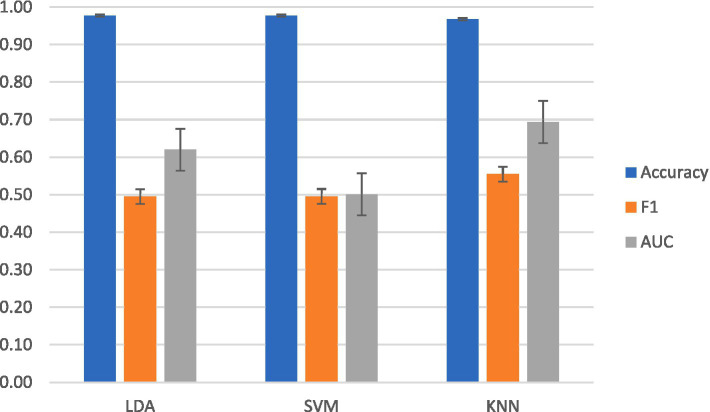
Averaged intrasubject classification results across classifier models.

Performance across subjects was plotted for KNN in Figure 4. The information transfer rate for KNN was 5.07 bits per trial, yielding a rate of 304.15 bits per minute.

**Figure 5:**
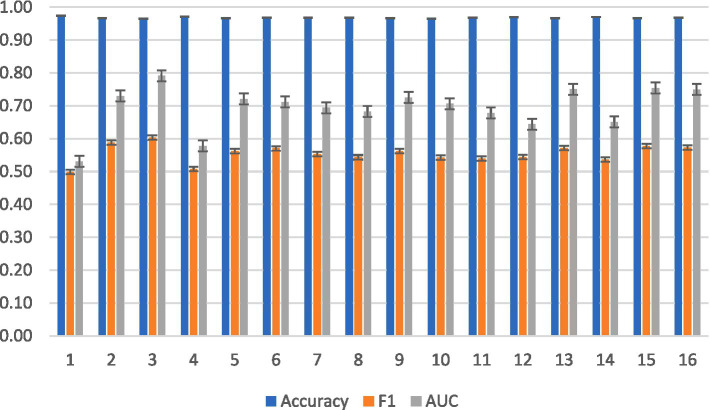
Averaged intrasubject classification results for KNN across subjects.

None of the subjects were BCI illiterate, as indicated by their high accuracy and F1 scores in Figure 5.

### 3.3 Intersubject classification

**Figure 6:**
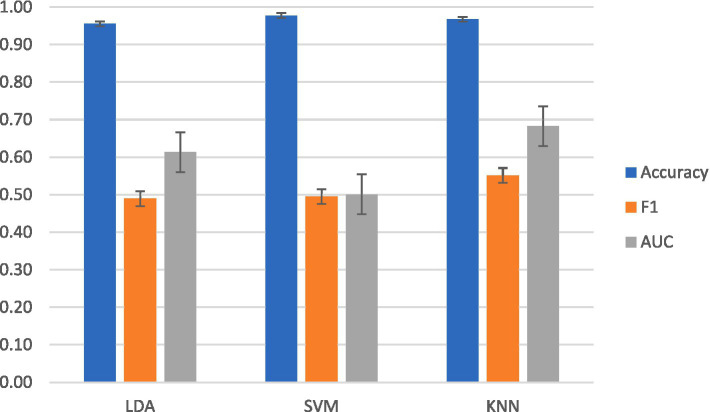
Averaged results from intersubject classification across classifier models.

In terms of accuracy, SVM was the highest-performing classifier for intersubject classification. SVM achieved a mean accuracy of 98 ± 0.01%, a mean F1 score of 0.50 ± 0.02, and a mean AUC-ROC of 0.50 ± 0.003. As shown in Figure 6, the highest mean F1 and AUC-ROC scores were with KNN at mean accuracy of 97 ± 0.001%, a mean F1 of 0.55 ± 0.01, and a mean AUC-ROC of 0.68 ± 0.002. LDA achieved a mean accuracy of 98 ± 0.002%, a mean F1 score of 0.49 ± 0.03, and a mean AUC-ROC of 61 ± 0.002.

ITR has been calculated in prior comparable BCIs (LaRocco and Paeng, 2020), and the ITR was calculated for a phoneme-based EEG BCI. The information transfer rate for SVM and LDA was 5.07 bits per trial, yielding a rate of 304.15 bits per minute.

### 3.4 Top features

Based on the average maximum distances between phonemes, gamma spectral power was the most consistent feature between individual phonemes among the 125 highest reliably separable feature types. Beta band power and amplitude were also consistent between the 44 phonemes. Channels F7 and F3 consistently showed the most significant variances between phonemes.

## 4 Discussion

### 4.1 Summary

Data from all 16 participants show that phoneme-based classification is a viable system for EEG BCI. Using open-source hardware and software, all 44 phonemes of the English language were correctly identified in the vast majority of intrasubject and intersubject classification cases at a maximum average accuracy of 98% with SVM. No prior work used a complete phoneme set for a language (Panachakel et al., 2021). The maximum averaged F1 score was 0.55 ± 0.01 and an AUC-ROC of 0.68 ± 0.002 with KNN. Using the correct data, even older machine learning algorithms can reliably perform phoneme classification. The dominant EEG features allowing correct classification of phonemes included gamma band power on channels F3 and F7, corroborating previous studies suggesting language-forming regions of the brain are most active (Jahangiri and Sepulveda, 2019; Wang et al., 2021). These speech-forming regions include Broca’s region and the frontal lobes (Trans Cranial Technologies, 2012; Pan et al., 2021; Wang et al., 2021; Lopez-Bernal et al., 2022). Potential users must be fluent in English, have normal-to-corrected vision, and have functional hearing. Unlike evoked potential virtual keyboard systems, imagined speech BCIs do not require diversion from a task, making them more practical for real-world application. The template established here could be applied to other groups and languages to ensure reliable replication. While this study establishes a precedent, a follow-up iteration could improve it.

### 4.2 Limitations

The scope of BCI deployment limited this study. The highest average maximum F1 score, 0.55 ± 0.01, needs to be substantially improved for greater reliability. Future implementations could utilize specialized classifier ensembles to increase the separability of phonemes. Other feature selection methods and dimensionality reduction could be investigated to improve classifier outcomes. The lack of real-time feedback was the primary obstacle to practical use. While the classifier framework detailed was an offline BCI, a similar method was translated to online use (Sereshkeh et al., 2017b; Jahangiri and Sepulveda, 2019). The trial length of 1 s is substantially longer than human awareness and neural processes for lexical selection, decreasing the ease of use and ITR. In addition, a longer sample size could improve confidence in the generalizability of the results. These limitations can be overcome through iterative improvements in future studies.

### 4.3 Future work

The system should be applied to a real-time text-to-speech task to continue this research, directly adapting the one-versus-rest classifier approach by having each new feature set exposed to 44 phoneme-specific classifiers and a selecting the category corresponding to the highest prior accuracy, F1 score, and/or AUC-ROC (Sereshkeh et al., 2017b; Jahangiri and Sepulveda, 2019). The latency of human awareness is approximately 0.1 s, a tenth of the current trial length. Even decreasing the trial length by half, from 1 s to 0.5 s, could improve the ITR and ease of use (Wang et al., 2021). Testing a real-time phoneme EEG BCI in the context of a brain-to-brain interface (BBI) could assess its advantages in a performance-related task, potentially alongside non-visual and non-auditory feedback, such as haptics or direct electrical stimulation (LaRocco and Paeng, 2020; Nicolelis, 2022). Developing an intuitive phoneme-based EEG BCI with open-source hardware and software demonstrates the potential ease with which the technology could be deployed in real-world applications.

## 5 Conclusion

An imagined speech EEG BCI using open-source hardware and software allowed 16 participants to successfully and reliably identify all 44 phonemes of the English language. Combining spatiotemporal and amplitude features with machine learning models yielded a maximum average accuracy of 98% for intrasubject and intersubject classification. The most consistently unique features across phonemes were gamma band activity on F3 and F7, aligning with prior research (Jahangiri and Sepulveda, 2019). The maximum average F1 score, 0.55 ± 0.01, should be increased to ensure reliability. However, adjustments to feature selection, trial window length, and classifier algorithms may improve performance. In summary, further iterative changes should be made to this method, which is directly deployable in commercially available systems and software.

## Data availability statement

The datasets presented in this study can be found in online repositories. The names of the repository/repositories and accession number(s) can be found at: https://github.com/javeharron/ghostTalkerAlpha.

## Ethics statement

The studies involving humans were approved by Ohio State University IRB 2023H0194. The studies were conducted in accordance with the local legislation and institutional requirements. The participants provided their written informed consent to participate in this study.

## Author contributions

JL: Conceptualization, Formal analysis, Funding acquisition, Methodology, Project administration, Writing – original draft. QT: Investigation, Resources, Supervision, Writing – review & editing. SL: Investigation, Project administration, Writing – review & editing. JM: Data curation, Investigation, Writing – review & editing. CH: Conceptualization, Methodology, Software, Writing – review & editing. SG: Data curation, Writing – review & editing.
